# Molecular detection of *Strongyloides* sp. in Australian Thoroughbred foals

**DOI:** 10.1186/s13071-021-04966-1

**Published:** 2021-09-03

**Authors:** Ghazanfar Abbas, Abdul Ghafar, Anson V. Koehler, Jenni Bauquier, Edwina J. A. Wilkes, Caroline Jacobson, Anne Beasley, John Hurley, Lucy Cudmore, Peter Carrigan, Brett Tennent-Brown, Charles El-Hage, Martin K. Nielsen, Charles G. Gauci, Kristopher J. Hughes, Ian Beveridge, Abdul Jabbar

**Affiliations:** 1 Melbourne Veterinary School, The University of Melbourne, Werribee, Australia; 2 School of Animal and Veterinary Sciences, Charles Sturt University, Wagga Wagga, Australia; 3 Centre for Animal Production and Health, Murdoch University, Murdoch, Australia; 4 School of Agriculture and Food Science, University of Queensland, Gatton, Australia; 5 Swettenham Stud, Nagambie, Australia; 6 Scone Equine Hospital, Scone, Australia; 7 M.H. Gluck Equine Research Center, Department of Veterinary Science, University of Kentucky, Lexington, KY USA

**Keywords:** Australian Thoroughbred horses, *Strongyloides* sp., Genetic characterisation, Sanger sequencing

## Abstract

**Background:**

*Strongyloides westeri* is found in the small intestine of young horses, mainly in foals up to about 16 weeks of age. The main source of infection for foals is through transmammary transmission, and foals can develop acute diarrhoea, weakness, dermatitis and respiratory signs. The epidemiology of *S. westeri* in Australia is largely unknown. Further, molecular techniques have never been employed for detection of *S. westeri* in horses. This pilot study aimed to assess the utility of a molecular phylogenetic method for the detection of *S. westeri* in the faeces of foals.

**Methods:**

Faecal samples were collected from a foal of less than 2 months of age, and eggs of *Strongyloides* sp. were detected using the modified McMaster technique. DNA was extracted from purified eggs, and a partial fragment of the small subunit of the nuclear ribosomal DNA (18S) was characterised using polymerase chain reaction, DNA sequencing and phylogenetic methods.

**Results:**

Microscopic examination of faeces revealed small ellipsoidal eggs typical of *Strongyloides* sp. The 18S sequence generated by PCR in this study revealed 98.4% identity with that of a reference sequence of *S. westeri* available from GenBank. Phylogenetic analyses revealed a polyphyletic clustering of *S. westeri* sequences.

**Conclusion:**

This is the first study reporting the detection of DNA of *Strongyloides* sp. in faeces of a foal using a molecular phylogenetic approach targeting the variable region of 18S rDNA. It is anticipated that this study will allow future molecular epidemiological studies on *S. westeri* in horses.

**Graphical abstract:**

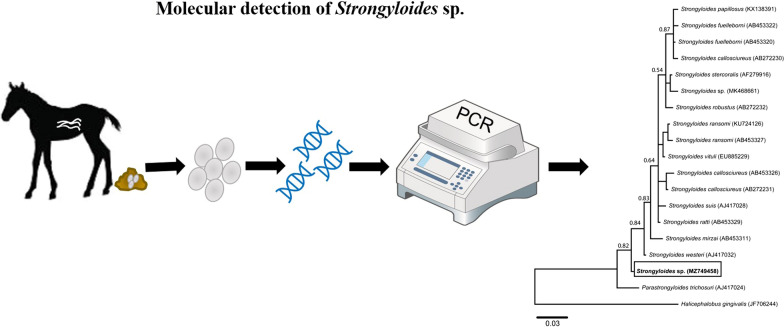

**Supplementary Information:**

The online version contains supplementary material available at 10.1186/s13071-021-04966-1.

## Background

*Strongyloides* (threadworms) is a genus of gastrointestinal nematodes of vertebrates comprising more than 50 species [1]. *Strongyloides westeri* infects horses, mainly young foals up to 16 weeks of age, and it is the first nematode to reach patency in the small intestine of foals [2]. A remarkable feature of the *S. westeri* life cycle is the ability to alternate between a sexual free-living phase and a parasitic phase consisting only of adult females shedding eggs by parthenogenesis [2].

Foals may become infected with parasitic third-stage larvae (L3s) of *S. westeri* through transmammary, percutaneous and oral routes. However, the transmammary route is considered the main source of transmission where L3s are transmitted from mare to foal via milk, which starts as early as day 4 postpartum and may continue up to several weeks [2–4]. The prepatent period via transmammary transmission is slightly shorter (8–12 days) than those of percutaneous and oral routes (10–14 days) because larvae migrating via the mammary glands are at an advanced stage of development and appear to not undergo somatic migration in the foals. [5].

Depending upon the route of transmission and parasite burden, foals can develop acute diarrhoea, weakness, dermatitis and respiratory signs [3, 6]. Episodes of frenzied foal behaviour are characterised by skin irritation, extreme discomfort and dermatitis due to infective L3s [7]. A significant association between high faecal *S. westeri* egg counts and diarrhoea in younger foals has been reported [8].

The modified McMaster faecal egg count (FEC) technique has been the most commonly used method for the diagnosis of *S. westeri* in horses. Some studies have reported total worm counts based on recovery of adult worms and larvae at necropsy [2, 9–13]. Although sensitive and advanced molecular tools have been used for the detection of other *Strongyloides* spp. from humans and animals [14–18], one reason that very little is known about the molecular epidemiology of *S. westeri* is that such tools have never been employed for its detection in horses. Additionally, data on the genetic diversity within this species are scarce, as only a single partial sequence of the small subunit of nuclear ribosomal DNA (18S) is available in the public database, GenBank [19]. Therefore, this study aimed to assess the utility of a molecular phylogenetic method for the detection of *S. westeri* in the faeces of foals by amplifying the partial 18S sequence.

While conducting a national survey on the gastrointestinal nematodes of Thoroughbred horses in Australia, we identified faecal samples (collected at a monthly interval) of a foal positive for *Strongyloides* sp. eggs during routine screening. These samples came from a Thoroughbred farm in the Hunter Valley, New South Wales, Australia, which has warm and temperate climatic conditions, with an average annual rainfall of 630 mm. The farm had a total of 335 horses, including 86 resident mares, 73 yearlings and 60 foals. All young horses were dewormed routinely every 7–8 weeks, while the adult horses receive anthelmintics preventatively every 4 to 6 months. Currently, no pre-foaling deworming of mares is performed on this farm, and mares are dewormed using a combination of moxidectin and praziquantel (Equest^®^ Plus Tape, Zoetis Pty. Ltd., Australia) 10 days after foaling, whereas foals receive a combination of oxfendazole and pyrantel (Strategy-T^®^, Virbac Pty. Ltd., Australia) at 2 months of age.

Following the detection of *Strongyloides* sp. eggs, fresh faecal samples were collected from a foal at the age of 23 and 52 days and shipped overnight to the laboratory. Each faecal sample was tested using the modified McMaster technique [20]. Briefly, four grams of faeces were mixed with four ml of water. The faecal slurry was then mixed with 52 ml of sucrose (specific gravity 1.27, www.csrsugar.com.au) solution and homogenised using a spatula. Following homogenisation, a sample was pipetted into two chambers of a Whitlock egg counting slide (www.whitlock.com.au). After 10 min, eggs were counted under a compound light microscope. The minimum detection limit using this method was 15 eggs per gram (epg) of faeces.

Five ml of the remaining suspension containing nematode eggs and saturated sugar solution from the sample were transferred to a 50-ml Falcon tube for recovery and concentration of nematodes eggs as previously described [21]. The washed eggs in each sample were transferred to a microcentrifuge tube and stored at −20 °C until further use. Following thawing, 250 µl of the concentrated egg solution were used to extract and isolate DNA using the DNeasy PowerSoil extraction kit (Qiagen, Hilden, Germany) according to the manufacturer’s protocol. DNA samples were stored at −20 °C until further molecular analysis.

A molecular phylogenetic method was used for the unequivocal identification of *Strongyloides* sp. eggs present in foal faeces from the farm under investigation. The partial 5′ variable region (392 base pairs) of the 18S DNA was amplified using polymerase chain reaction (PCR) in a T100 thermal cycler (BioRad, Hercules, CA, USA) using SSUA_F (5′-AAAGATTAAGCCATGCATG-3′) and SSU22_R (5′-GCCTGCTGCCTTCCTTGGA-3′) primers as previously described by Dorris et al. [19]. PCR amplifications (initial denaturation at 95 °C for 5 min followed by 35 cycles of denaturation at 95 °C for 30 s, annealing at 60 °C for 20 s and extension at 72 °C for 30 s, and the final extension at 72 °C for 5 min) were carried out in a final reaction volume of 25 µl containing 3.12 mM of each deoxynucleotide triphosphate (dNTP), 12.5 pmol of each primer, 10 mM Tris–HCl (pH 8.4), 75 mM MgCl_2_ and 0.62 U of GoTaq Flexi DNA polymerase (Promega, Madison, WI, USA). Known positive (genomic DNA of *Strongyloides stercoralis*) and negative (Milli-Q H_2_O) controls were included in each PCR run. Aliquots (5 μL) of individual amplicons were analysed on 1.5% (w/v) agarose gel in Tris–borate-EDTA buffer stained with GelRed (Biotium) and visualised using a GelDoc system (Bio-Rad, Hercules, CA, USA).

Amplicons were purified using shrimp alkaline phosphate and exonuclease I (Thermo Fisher Scientific, Australia) before automated Sanger DNA sequencing using the PCR primers in separate reactions. The quality of the sequences was assessed in the Geneious Prime 2021.1.1 software (Biomatters Ltd., Auckland, New Zealand; www.geneious.com). The DNA sequence determined herein has been submitted to the GenBank database under the accession number MZ749458. Published 18S sequences of *Strongyloides* spp. from humans and domestic and wild animals were obtained from GenBank and were aligned with our 18S sequence using MUSCLE in Mesquite v.3.61 (http://www.mesquiteproject.org) using default settings and were trimmed to uniform lengths of 306 bp. The evolutionary model (T92 + G) of the DNA sequence data set was determined using the Akaike and the Bayesian information criteria (AIC and BIC) tests in jModelTest v.2.1.5 [22]. Neighbour-joining (NJ) trees were constructed using MEGA 10.2.5 [23], and Bayesian inference (BI) trees were built using MrBayes software [24]. The NJ trees were constructed with 10,000 bootstrap replicates using the Tamura–Nei distance method. The BI analysis was run for 20,000,000 generations (ngen = 20,000,000) to calculate posterior probabilities (pp), with two runs, with every 200th tree saved (samplefreq = 200). The 18S rDNA sequence of *Halicephalobus gingivalis* was used as an outgroup. Tree topology was checked for consensus between NJ and BI analyses.

**Fig. 1:**
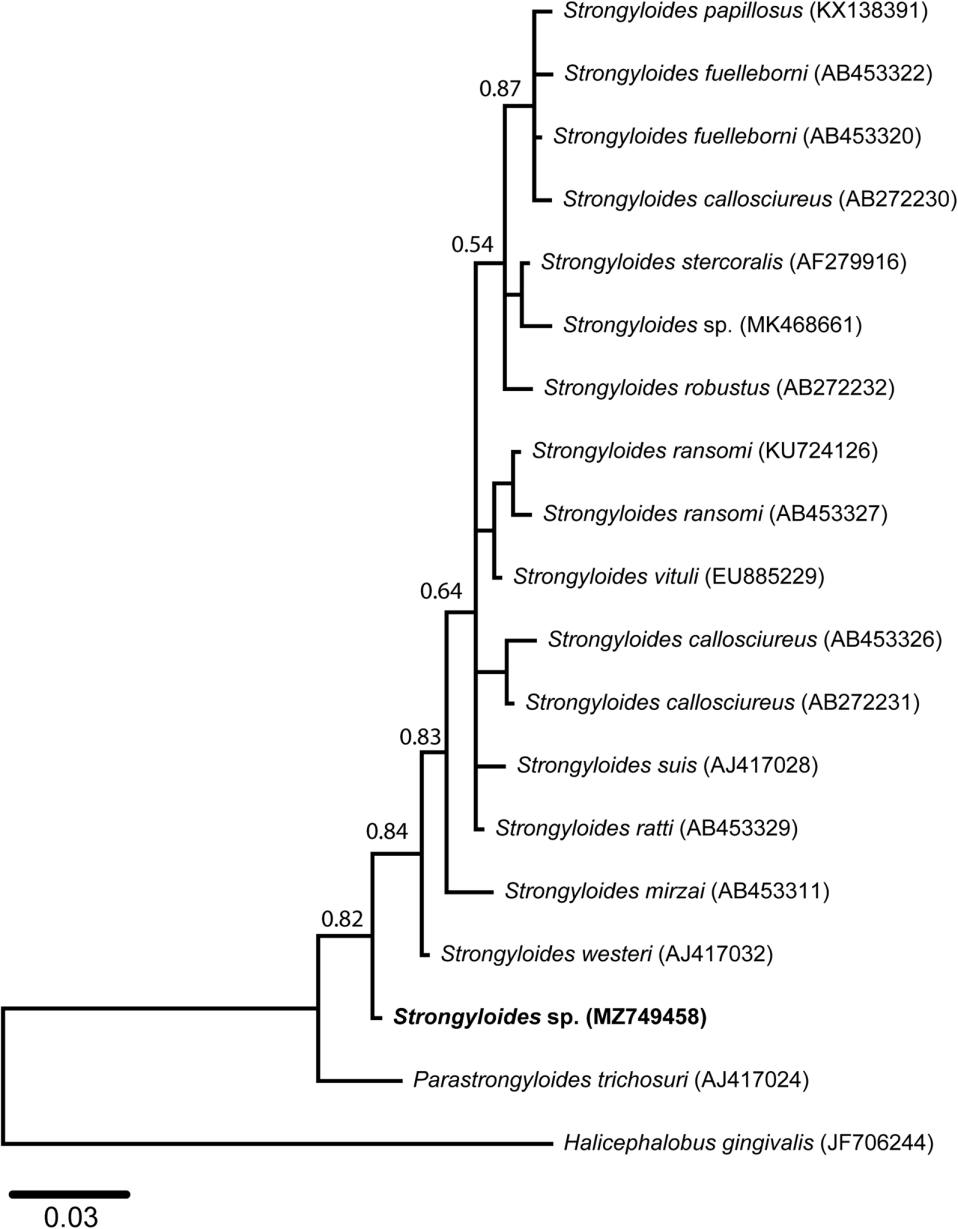
Genetic relationship of the small subunit nuclear ribosomal DNA sequence of *Strongyloides* sp. collected form Thoroughbred horses in Australia determined in this study. The relationships were inferred based on phylogenetic analyses of the 18S data using Bayesian inference (BI) and distance-based neighbour-joining (NJ) methods. *Halicephalobus gingivalis* was used as the outgroup. There was a concordance in the topology between this BI tree and that produced using the NJ method (not shown). Nodal support is given as a posterior probability for BI, and bootstrap values for NJ were below 50%, hence not provided on this tree. The scale bar indicates the number of inferred substitutions per nucleotide site

Microscopic examination revealed small ellipsoidal nematode eggs ranging between 40–50 × 50–60 µm, and FECs were 90 and 750 epg in samples collected at the age of 29 and 52 days, respectively. PCR amplicons revealed an expected size band (~ 400 bp) on 1.5% agarose gel, and the partial 18S DNA sequence (363 bp) obtained herein (GenBank accession no. MZ749458) had a 98.4% similarity with that of a reference sequence identified from *S. westeri* in the USA (GenBank accession no. AJ417032) [19]. Pairwise comparison of our sequence with selected 18S reference sequences of various *Strongyloides* spp. showed percentage differences ranging between 1.6 and 5.7% (Table 1). An alignment of the 18S reference sequences of *Strongyloides* spp. together with our sequence revealed four nucleotide differences with *S. westeri* linked to transition (C ↔ T; position 40) or transversions (A ↔ T; positions 41, 82, and 88) (see Additional file 1: Figure S1). Genetic relationships assessed utilising NJ and BI methods produced similar topologies; therefore, only the BI tree is presented here (Fig. 1). The phylogenetic analyses revealed that the 18S sequence of *Strongyloides* sp. determined herein grouped outside all other *Strongyloides* spp. included in this study (pp = 0.84). However, our 18S sequence grouped between those of the only available reference sequence of *S. westeri* (GenBank AJ417032, fixed material from horse) and *Parastrongyloides trichosuri* (AJ417024, fresh material from the Australian brushtail possum) [19].

**Table 1:** Pairwise comparison of percent differences of 18S SSU rDNA sequence determined herein (bold) and the selected reference sequences of *Strongyloides* spp.

Taxa no.	Strongyloides species (GenBank accession no.)	1	2	3	4	5	6	7	8	9	10	11	12	13	14	15	16	17	18
1	***Strongyloides*sp. (MZ749458)**	–																	
2	*S. westeri* (AJ417032)	1.6	–																
3	*S. robustus* (AB272232)	3.8	3	–															
4	*S. stercoralis* (AF279916)	3.8	2.3	2.3	–														
5	*Parastrongyloides trichosuri* (AJ417024)	5.7	6.8	6	7.5	–													
6	*S. suis* (AJ417028)	3.4	1.9	3.4	3.4	8.3	–												
7	*S. vituli* (EU885229)	3.1	1.6	3	3	7.9	1.2	–											
8	*S. ransomi* (KU724126)	3.4	1.9	3	3	7.9	1.6	0.4	–										
9	*S. papillosus* (KX138391)	3.1	2.3	2.3	2.3	7.2	2.7	1.6	1.6	–									
10	*Strongyloides* sp. (MK468661)	3.4	1.9	2.3	1.5	7.2	3.1	2.7	2.7	2.3	–								
11	*S. mirzai* (AB453311)	3.8	2.3	3.8	3	8.6	3	2.3	2.7	2.3	3	–							
12	*S. ratti* (AB453329)	3.1	1.6	2.3	2.3	7.5	1.2	0.8	1.2	2.3	1.9	3	–						
13	*S. ransomi* (AB453327)	3.8	2.3	3.4	3.4	7.5	1.9	0.8	0.4	1.9	3.1	3	1.6	–					
14	*S. callosciureus* (AB453326)	4.9	3.4	2.7	3.4	7.9	3.1	2.7	3.1	3.4	3.4	4.2	1.9	2.7	–				
15	*S. fuelleborni* (AB453322)	3.8	3.1	2.3	2.3	7.5	3.4	2.3	2.3	0.8	2.3	3	2.3	2.7	3.4	–			
16	*S. fuelleborni* (AB453320)	3.4	2.7	1.9	1.9	7.2	3.1	1.9	1.9	0.4	1.9	2.7	1.9	2.3	3.1	0.4	–		
17	*S. callosciureus* (AB272231)	4.2	2.7	1.9	2.7	7.2	2.3	1.9	2.3	2.7	2.7	3.4	1.2	1.9	0.8	2.7	2.3	–	
18	*S. callosciureus* (AB272230)	3.1	2.3	2.3	2.3	7.5	2.7	1.6	1.9	0.8	2.3	2.3	1.6	2.3	2.7	0.8	0.4	1.9	–

This study describes the morphology of eggs and molecular detection of *Strongyloides* sp. in an Australian Thoroughbred foal. It reports the first PCR-based detection of DNA of *Strongyloides* sp. from eggs purified from faecal samples of a foal, which paves the way for future molecular epidemiological studies on *S. westeri* in horses. Previously, only one study has reported *S. westeri* in Australian horses, based on the examination of adult worms collected at necropsy [13].

*Strongyloides westeri* is believed to infect foals up to 4 months of age, and the foal tested in the present study was less than 2 months [2]. The route of infection of *S. westeri* found in this study is likely to be transmammary, as the mare did not receive a pre-foaling anthelmintic treatment, although we have not demonstrated the presence of larvae in the milk. Third-stage larvae of *S. westeri* have been reported in the milk of mares from the fourth day after foaling up to day 47 postpartum [3, 4]. Recently, a study demonstrated the presence of *S. westeri* in foals of up to 8 months of age, suggesting that infection could last longer in foals, weanlings, or yearlings, but the number of parasites declines as animals get older [12].

The prevalence for *S. westeri* in Australian horses is not well studied and could be higher than previously thought. During the first half of the twentieth century, *S. westeri* was highly prevalent in foals in central Kentucky and other parts of the world. However, from the mid-twentieth to early twenty-first centuries, the prevalence of this parasite declined markedly (< 10% prevalence of *S. westeri* in the surveyed horses) due to the use of highly effective anthelmintics, including benzimidazoles and macrocyclic lactones [9, 13]. However, *S. westeri* prevalence in foals from the same sampling area in Kentucky, USA, increased from < 6% (late 1900s and early 2000s) to 30% (2014) [11], probably due to the decreased use of ivermectin in foals because of the development of anthelmintic resistance in *Parascaris* spp. in horses [10]. In another study, lower prevalence and sporadic occurrence of *S. westeri* in foals from New Zealand was reported [25]. It is possible that the recommended shift away from frequent deworming of foals with macrocyclic lactones (due to the development of resistance in ascarid worms) may lead to an increase in *S. westeri* prevalence. A molecular phylogenetic technique, such as that described herein, would be an important tool for assessing the occurrence of *S. westeri* in horses worldwide.

To date, all studies of *S. westeri* have used FEC and larval or adult morphology following coproculture for the identification of parasites, which both have lower sensitivity than PCR [26, 27]. Further, the identification of *Strongyloides* spp. solely on the basis of morphological characteristics of different stages of parasites can be difficult [17]. More than 50 species of *Strongyloides* have been reported from various hosts [1], and the list is expanding, as recently a new species *S. vituli* of cattle was separated from *S. papillosus,* although previously both were included under a single species name [16, 28].

Comparison of the partial 18S sequence of *Strongyloides* sp. determined in the current study and that of *S. westeri* (GenBank AJ417032) from the USA revealed a difference of four base pairs over a sequence length of 270 bp (see Additional file 1: Figure S1) which is an unlikely finding in the same species given the high degree of conservation of 18S in the genus *Strongyloides* [19]. This difference could be due to (i) a rare polymorphism (variation at a single position in a DNA sequence among individuals) among different copies of SSU loci within genomes of *Strongyloides* spp. as well as other nematodes [19, 29] and/or (ii) the difference in the quality of DNA used as a template in PCRs. For example, we extracted genomic DNA from eggs purified from freshly collected faecal samples from a foal, while Dorris et al. [19] extracted DNA from a formalin-fixed specimen of *S. westeri*. Previously, a few studies reported a difference of 10% in DNA sequences from formalin-fixed specimens versus fresh specimens of the same species [30]. Dorris et al. [19] also proposed the possibility of a non-random effect on DNA due to formalin fixation that could cause a repeatable change in subsequent sequences. Similarly, Eberhardt et al. [28] found a difference of four base pairs between 18S sequences of *S. papillosus* and those of the same species previously published by Dorris et al. [19], and it was hypothesised that these differences could possibly be due to molecular synapomorphy (characters shared by a group of taxa due to their inheritance from a common ancestor) which exists in the 330-bp 18S sequence used for phylogenetic analyses by Dorris et al. [19].

In the present study, phylogenetic analyses revealed that the two possible *S. westeri* sequences did not group together (see Fig. 1) which could potentially be due to soft polytomy (insufficient phylogenetic information due to short sequence length) or sequences belonging to separate species. Previously, Dorris et al. [19] compared the SSU sequences of 12 species of *Strongyloides* and related nematodes and found that the *S. westeri* sequence showed a separate basal polytomy from the rest of the closely related species in *Strongyloides* that branched together in two clades. Previously, *S. stercoralis* was considered as a single species known to infect dogs; however, based on the analyses of the hypervariable region of 18S and cytochrome *c* oxidase subunit I mitochondrial DNA, two genetically different variants of *S. stercoralis* were identified which can infect dogs and humans [31]. Likewise, *S. fuelleborni* known to infect humans and other primates, has now been divided into two subspecies, *S. fuelleborni fuelleborni* and *S. fuelleborni kellyi*, on the basis of molecular evidence [32]. Similar diversification is observed in *S. callosciureus,* a species that infects a variety of squirrels worldwide [17]. It has been proposed that the species diversity in *Strongyloides* genus may be associated with the geographical location and evolutionary history of the hosts [17], including horses, which requires further investigation.

## Conclusion

This is the first study reporting the detection of DNA of *Strongyloides* sp. in the faecal samples of a Thoroughbred foal using a molecular phylogenetic approach targeting the variable region of 18S rDNA. Phylogenetic analyses revealed a basal polytomy of the sequence determined here, and it grouped separately along with the only reference sequence of *S. westeri*. We hypothesise that this genetic variation could be due to (i) the genetic diversity within 18S rDNA of *S. westeri*, (ii) a polymorphism, (iii) sequencing errors in the previously reported sequence from the formalin-fixed specimen of *S. westeri* or (iv) the existence of cryptic or subspecies. However, these hypotheses warrant further molecular analyses of a range of DNA samples extracted from various developmental stages of the *S. westeri* collected from different locations globally using multiple molecular markers. The use of molecular phylogenetic tools offers opportunity for improved diagnosis and epidemiological studies of *S. westeri* in horses worldwide.

## Supplementary Information

**Additional file 1:Figure S1.** Alignment of the small subunit nuclear ribosomal DNA (18S) sequence of *Strongyloides* sp. determined herein (bold) and the selected reference sequences. A dot indicates an identical nucleotide with respect to the sequence of Strongyloides sp.; a dash indicates an insertion/deletion (indel) event.

## Data Availability

All data generated or analysed during this study are included in this published article.
