# Supplementary material for: Molecular detection of Strongyloides sp. in Australian Thoroughbred foals
Source: Parasit Vectors. 2021 Sep 3;14:444. doi: 10.1186/s13071-021-04966-1 (PMC8414860; doi:10.1186/s13071-021-04966-1)
Supplement: Supplementary file 1 — Additional file 1:Figure S1. Alignment of the small subunit nuclear ribosomal DNA (18S) sequence of Strongyloides sp. determined herein (bold) and the selected reference sequences. A dot indicates an identical nucleotide with respect to the sequence of Strongyloides sp.; a dash indicates an insertion/deletion (indel) event. [file 13071_2021_4966_MOESM1_ESM.docx]

10 20 30 40 50 60 70 80 90 100

....|....|....|....|....|....|....|....|....|....|....|....|....|....|....|....|....|....|....|....|

**MZ749458 *Strongyloides* sp.** **GTTGGATAACTGAGGTAATTCTTGAGCTAATACACGCTTCATATACCACATTAGTGGTGCGTTTATTTGATTAAACC-A--ATTTA-ATGGTTGACTCAA**

AJ417032 *S. westeri* .......................................TT....................................-.--T....-T............

AB272232 *S. robustus* ......................................A.T...G................................A.--T.A..-TC...........

AF279916 *S. stercoralis* ......................................ATT....................................-.TTT.A..-T............

AJ417024 *Parastrongyloides trichosuri* ...........................................AG.......C........................A.----A..-.............

AJ417028 *S. suis* ..A....................................TT....................................-.--T...T-TC...........

EU885229 *S. vituli* .......................................TT....................................-.--T...T-TC...........

KU724126 *S. ransomi* .......................................TT....................................-.--T.C.T-TC...........

KX138391 *S. papillosus* ......................................ATT....................................-.--T.A..-.C...........

MK468661 *Strongyloides* sp. ......................................GTTA...................................-.--T.A..-T............

AB453311 *S. mirzai* ..G......A............................ATT....................................-.--T....TT............

AB453329 *S. ratti* .......................................TT....................................-.--T...T-TC...........

AB453327 *S. ransomi* .......................................TT...........C........................-.--T.C.T-TC...........

AB453326 *S. callosciureus* .....TA...............................ATT...G.......C........................-.--T...T-TC...........

AB453322 *S. fuelleborni* .............C........................ATT....................................-.--T.A..-.C...........

AB453320 *S. fuelleborni* ......................................ATT....................................-.--T.A..-.C...........

AB272231 *S. callosciureus* ......................................ATT...G.......C........................-.--T...T-TC...........

AB272230 *S. callosciureus* ......................................ATT....................................-.--T....-.C...........

110 120 130 140 150 160 170 180 190 200

....|....|....|....|....|....|....|....|....|....|....|....|....|....|....|....|....|....|....|....|

**MZ749458 *Strongyloides* sp.**  **AATATCCTCGCTGATTTTATTATTAAAACATACCGTATGTGTATCTGGTTTATCAACTTTCGATGGTAGGGTATTGGCCTACCATGGTTGTGACGGATAA**

AJ417032 *S. westeri* ....................................................................................................

AB272232 *S. robustus* ..................G...C.............................................................................

AF279916 *S. stercoralis*  ..................G...C.............................................................................

AJ417024 *Parastrongyloides trichosuri* .................--...AC.......................C........................................GT..........

AJ417028 *S. suis* ..................G.................................................................................

EU885229 *S. vituli* ........T.........G.................................................................................

KU724126 *S. ransomi* ........T.........G.................................................................................

KX138391 *S. papillosus* ........T.........G.................................................................................

MK468661 *Strongyloides* sp. ..................G...C.............................................................................

AB453311 *S. mirzai* ........T.........G.................................................................................

AB453329 *S. ratti* ..................G...C.............................................................................

AB453327 *S. ransomi* ........T.........G.................................................................................

AB453326 *S. callosciureus* ..................G...C.............................................................................

AB453322 *S. fuelleborni* ........T.........G...C.............................................................................

AB453320 *S. fuelleborni* ........T.........G...C.............................................................................

AB272231 *S. callosciureus* ..................G...C.............................................................................

AB272230 *S. callosciureus* ........T.........G...C.............................................................................

210 220 230 240 250 260 270

....|....|....|....|....|....|....|....|....|....|....|....|....|....|

**MZ749458 *Strongyloides* sp. CGGAGAATTAGGGTTCGACTCCGGAGAGGGAGCCTGAGAAACGGCTACCACATCCAAGGAAGGCAGCAGG**

AJ417032 *S. westeri* ......................................................................

AB272232 *S. robustus* ......................................................................

AF279916 *S. stercoralis* ......................................................................

AJ417024 *Parastrongyloides trichosuri* ...G..................................................................

AJ417028 *S. suis* ..................................................A...................

EU885229 *S. vituli* ......................................................................

KU724126 *S. ransomi* ......................................................................

KX138391 *S. papillosus* ......................................................................

MK468661 *Strongyloides* sp. ......................................................................

AB453311 *S. mirzai* ......................................................................

AB453329 *S. ratti* ......................................................................

AB453327 *S. ransomi* ......................................................................

AB453326 *S. callosciureus* ......................................................................

AB453322 *S. fuelleborni* ......................................................................

AB453320 *S. fuelleborni* ......................................................................

AB272231 *S. callosciureus* ......................................................................

AB272230 *S. callosciureus* ......................................................................

**Additional File 1: Fig. S1.** Alignment of the small subunit nuclear ribosomal DNA (18S) sequence of *Strongyloides* sp. determined herein **(bold)** and the selected reference sequences. A dot indicates an identical nucleotide with respect to the sequence of *Strongyloides* sp.; a dash indicates an insertion/deletion (indel) event.
